# Effect of Heat Treatment on Microstructure and Tensile Property of Laser-Powder-Bed-Melted Al–Mn–Mg–Sc–Zr Alloy

**DOI:** 10.3390/ma18071638

**Published:** 2025-04-03

**Authors:** Zhiqiang Cao, Hui Yin, Jin Jiang, Mingliang Cui, Hao Zhang, Sheng Cao

**Affiliations:** 1 Center for Instrumental Analysis, University of Shanghai for Science and Technology, 516 Jungong Road, Shanghai 200093, China; caozhiqiang@usst.edu.cn; 2 Deyang Wanhang Die Forging Co., Ltd., China National Erzhong Group Co., Deyang 618000, China; heavenmilk@163.com (H.Y.); cuiminglianghost@163.com (M.C.); 3 China State Shipbuilding Co., Ltd., Huangpu Wenchong Shipbuilding Company Ltd., Guangzhou 510715, China; jiang142058@163.com; 4 College of Materials Science and Engineering, Beijing University of Technology, Beijing 100080, China; 5 Institute of Advanced Wear & Corrosion Resistant and Functional Materials, Jinan University, Guangzhou 510632, China; 6 College of Chemistry and Materials Science, Jinan University, Guangzhou 511443, China

**Keywords:** Al–Mn–Mg–Sc–Zr alloy, laser powder bed fusion, heat treatment, mechanical property

## Abstract

This study explored the effects of T5 and T6 heat treatments on the microstructure and tensile properties of a laser powder bed fusion (LPBF)-fabricated Al–Mn–Mg–Sc–Zr alloy. The as-built condition exhibited a bi-modal grain structure of equiaxed and columnar grains. Specimens after T5 heat treatment also had a bi-modal microstructure with slight grain growth and the precipitation of secondary Al_3_Sc, which enhanced the yield strength via precipitation hardening but reduced ductility. In contrast, T6 treatment triggered recrystallization, and the microstructure was only coarse equiaxed α-Al grains. This microstructure change was accompanied by coarsened primary Al_3_X and Al_6_(Mn, Fe) precipitates, partial Mg_2_Si dissolution, and significant secondary Al_3_Sc particle growth. Consequently, T6-treated specimens showed lower strength than their T5 counterparts and the poorest ductility due to brittle fracture induced by the stress concentration effect of coarse precipitates at grain boundaries.

## 1. Introduction

Additive manufacturing (AM) is known for offering a high degree of design freedom and producing customized products efficiently [1]. Among different AM techniques, laser powder bed fusion (LPBF) is a bottom-up layer-by-layer manufacturing technique using a fine high-energy laser beam (diameter: dozens of μm to 100 μm) to melt and fuse metal powder particles according to the sliced Computer-Aided Design (CAD) profile. It produces near-net-shape parts with optimized and integrated functions to meet the requirements of stringent applications in the military, aerospace, electronics, and automotive industries [2].

In recent years, the growing industrial demand for improved energy efficiency and enhanced engineering reliability has further highlighted the advantages of the LPBF process [3]. These benefits are amplified when utilizing high-strength aluminum (Al) alloys, known for their light weight, high thermal conductivity, excellent corrosion resistance, and superior mechanical properties [4,5]. Currently, the Scalmalloy^®^, a Sc/Zr-modified 5xxx alloy [6], marked the first milestone in the creation of high-strength Al alloys by LPBF. The precipitation of primary Al_3_X (X = Sc and/or Zr) nuclei, which are coherent with the Al matrix, during solidification induces the formation of equiaxed grains in LPBF fabricated Scalmalloy [7]. After heat treatment, a tensile yield strength exceeding 500 MPa has been reported [8], significantly surpassing that of most conventional Al alloys prepared by LPBF. In addition, a number of Sc/Zr-modified high-strength Al alloys that are particularly suitable for the LPBF process have been developed, such as Al–Mg–Sc–Zr [9,10,11,12], Al–Mn–Mg–Sc–Zr [13,14], Al–Fe–Sc–Zr [15], Al–Li–Sc–Zr [16], Al–Cu–Sc–Zr [17] and Al–Zn–Mg–Sc–Zr [18]. Among these alloys, the Al–Mn–Mg–Sc–Zr alloy (with the addition of supersaturated Mn, Sc, and Zr) demonstrated excellent printability with no cracks, a high ultimate tensile strength of 570 MPa, and an exceptional work-hardening behavior after a post-process heat treatment [19].

The post-process heat treatment is an important step in LPBF, which can tailor the microstructure, release the residual stress, and enhance the strength [20]. For Al alloys, the conventional T5 artificial aging (AA) heat treatment effectively eliminates residual stress while enhancing material strength without compromising ductility [21]. Notably, T5 treatment preserves the characteristic original microstructure while promoting the precipitation of strengthening phases, thereby improving hardness and tensile strength [19,22]. Some studies on LPBF-prepared Al–Mn–Mg–Sc–Zr alloy alloys have confirmed [20,23] that it reaches the peak hardness, the highest hardness, the greatest yield strength, the ultimate tensile strength, and the greatest elongation at break when artificial aging at 300 °C was applied for 5 h (T5). To be more specific, a recent study demonstrated that LPBF-produced Al–Mn–Mg–Sc–Zr alloy achieved a strength–ductility balance after T5 heat treatment, with an ultimate tensile strength of 528 MPa and an elongation of 14.5% [20]. However, further aging for 7 h led to the growth of Al_6_(Mn, Fe) precipitates, resulting in reduced ductility. Therefore, the control of artificial aging parameters is crucial in designing the microstructure and thereby affects the overall mechanical performance.

In contrast, the T6 heat treatment (solution heat treatment (SHT) followed by AA) removes the melt pool structure, resulting in significant grain growth to equiaxed morphology and leading to a more homogeneous microstructure [24]. After T6 heat treatment, the anisotropy of the LPBF-fabricated microstructure is reduced, and residual stress is completely relieved. In the subsequent AA stage, the precipitation of strengthening phases contributes to strength enhancement [25]. Consequently, T6 heat treatment achieves balanced mechanical performance by improving the toughness and ductility without compromising yield strength while simultaneously enhancing fatigue resistance [24,26]. Compared to the Al–Mn series alloys [27,28], LPBF-fabricated Al–Mn–Mg–Sc–Zr is known for reduced microstructural anisotropy because of grain-refining effects from the incorporation of Sc and Zr. However, the effect of T6 heat treatments on the microstructure and properties of LPBF-produced Al–Mn–Mg–Sc–Zr alloys is still not fully understood.

Therefore, this study investigated the relationship between the microstructure and the tensile properties of LPBF-fabricated high-strength Al–Mn–Mg–Sc–Zr alloys in the as-built (AB), as well as post-processed, T5 and T6 in heat-treated conditions. Microstructure was investigated by scanning electron microscope (SEM) and scanning transmission electron microscope (STEM)–high-angle annular dark field (HAADF) (STEM-HAADF). Properties were investigated by hardness and tensile tests. The relationships of heat treatment, microstructure and property were discussed.

## 2. Materials and Methods

### 2.1. Power Materials

**Figure 1:**
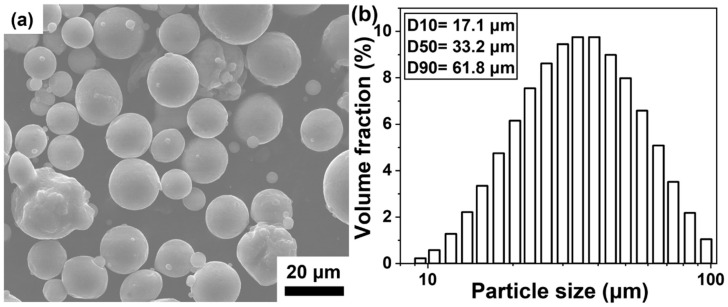
(**a**) Powder morphology and (**b**) particle size distribution of the studied alloys.

The raw powders for LPBF fabrication were Al–Mn–Mg–Sc–Zr powders prepared by a vacuum induction-melting gas atomization (VIGA) facility at Foshan Chengfeng Material Technology Co., Ltd. (Guangdong, China). The chemical composition of the LPBF powder was determined by using inductively coupled plasma atomic emission spectroscopy (ICP-AES), as detailed in Table 1. The morphologies of the raw powder particles are illustrated in Figure 1a. Most powders exhibited a spherical morphology with some satellited particles. The particle size distribution was measured by using a Mastersizer 3000, and the result is presented in Figure 1b. The D10, D50, and D90 values were 17.1 μm, 33.2 μm, and 61.8 μm, respectively.

### 2.2. Specimen Preparation

Before LPBF printing, the powders were dried for 5 h at 100 °C in a vacuum. The LPBF fabrication was performed on an AmPro160 LPBF machine with a laser spot size of 75 μm, a preheated substrate at 100 °C, and a scanning angle rotation of 66.67° between adjacent layers. The optimal printing parameters for highly dense specimens (99.9%) were 350 W (laser power), 1300 mm/s (scanning speed), 0.14 mm (hatch spacing), and 0.03 mm (layer thickness). Two types of specimens were prepared: (1) cubic specimens with dimensions of 10 × 10 × 10 mm^3^ for microstructural analyses and (2) cylindrical specimens with dimensions of φ10 mm × 60 mm for tensile tests.

### 2.3. Heat Treatment

After LPBF, the specimens were treated by two different heat treatments: (1) T5 heat treatment: AA at 300 °C for 5 h followed by furnace cooling; (2) T6 two-step heat treatment: SHT at 520 °C for 0.5 h followed by water quenching and then aging at 180 °C for 10 h followed by furnace cooling (T6) at a rate of 1 °C/min.

### 2.4. Microstructural Characterization

Metallographic specimens were prepared by sectioning with a precision cutting machine, followed by grinding and polishing. These specimens were then etched for 15 s in Keller’s reagent (2.5 mL HNO_3_, 1.5 mL HCl, 1 mL HF, and 95 mL H_2_O). The microstructure and pores were analyzed by using an IMM 5000 optical microscope (OM) and a JSM-7200F SEM. The grain orientations and size distributions were analyzed through Electron Backscattered Diffraction (EBSD) on a JSM-1T500HR SEM. STEM-HAADF and high-resolution transmission electron microscope (HRTEM) analyses were performed on an FEI-Talos F200X microscope coupled with a Super-X EDS detector, operating at a voltage of 200 kV. The fast Fourier transformation (FFT) in HRTEM was obtained by using Gatan DigitalMicrograph^®^ 3.5 software. Electron-transparent disks for TEM analysis were prepared by a Struers TenuPol-5 twin-jet electro-polisher (Struers, Copenhagen, Denmark)with an electrolyte containing a mixture of 90% methanol and 10% perchloric acid at −30 °C and 25 V.

### 2.5. Mechanical Property Evaluation

Microhardness values were measured by a Vickers hardness tester with a load of 0.3 kg and a dwelling time of 15 s. Twenty indentations were performed to obtain average microhardness values for each condition.

Room-temperature tensile tests were conducted by using M12 cylindrical specimens with a gauge diameter of 6 mm and a gauge length of 30 mm according to the ASTM E8 standard [29]. Three vertically built replicates (loading direction parallel to build direction Z) of each specimen condition were performed on a GNT-50 machine equipped with a 10 mm extensometer, maintaining a constant crosshead speed of 1 mm/min, and three replicates were generally used in evaluating the tensile performance of metallic materials [9]. Post-tensile fractographic examinations were carried out on a JSM-7200F SEM.

## 3. Results and Discussion

### 3.1. Microstructure

**Figure 2:**
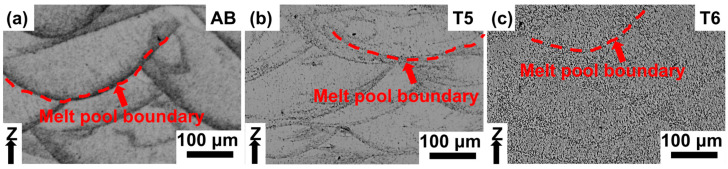
OM of Al–Mn–Mg–Sc–Zr specimens in different conditions. (**a**) As-built and (**b**) T5- and (**c**) T6-treated conditions.

Figure 2 presents the OM of LPBF-produced Al–Mn–Mg–Sc–Zr specimens in as-built and heat-treated conditions. The microstructure after T5 (Figure 2b) displayed crystal structures with a melt pool morphology similar to the AB condition (Figure 2a), with clearly defined melt pool boundaries. In contrast, the T6 specimens’ microstructure exhibited a gradual weakening of the melt pool boundary (Figure 2c), suggesting that the T6 treatment induces recrystallization in the microstructure and homogenization and promote a more uniform microstructure [30].

**Figure 3:**
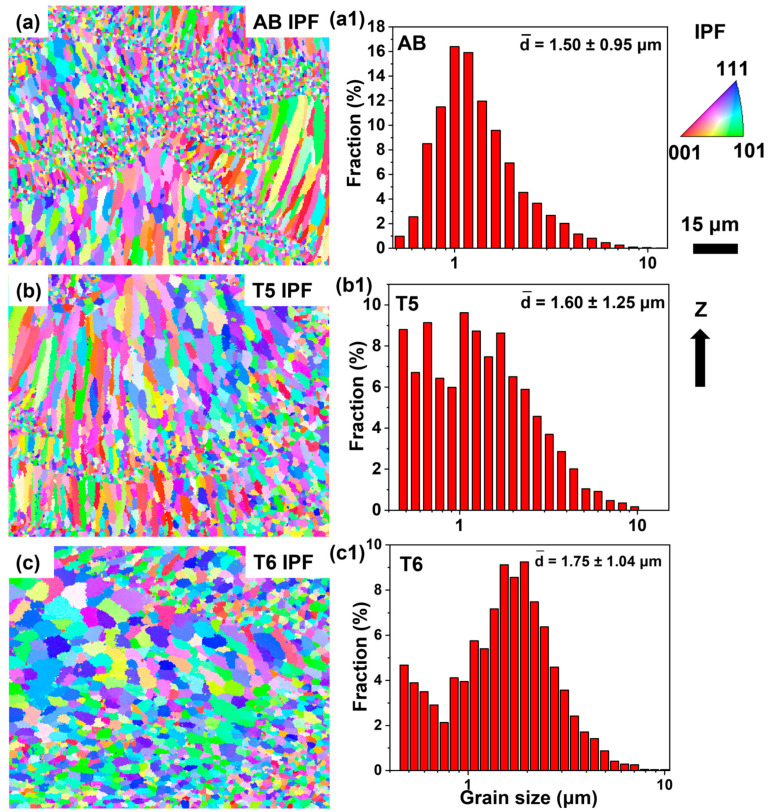
(**a**–**c**) EBSD–inverse pole figures (IPF) of the LPBF-fabricated Al–Mn–Mg–Sc–Zr specimens in the AB, T5, and T6 conditions; (**a1**–**c1**) the corresponding grain size distributions in (**a**,**b**).

EBSD analyses were conducted on these specimens. As shown in Figure 3a,b, the AB and T5 specimens had equiaxed grains (EGs) at the melt pool boundaries and columnar grains (CGs) within the melt pool. In contrast, the microstructure was recrystallized and comprised coarsened equiaxed grains after the T6 heat treatment, as shown in Figure 3c, and there were fine (recrystallized grains at the melt pool boundary) and coarse equiaxed grains (recrystallized and coarsened original columnar grains). Based on the EBSD results, the average grain sizes were determined to be 1.50 ± 0.95 μm, 1.60 ± 1.25 μm, and 1.75 ± 1.04 μm for AB, T5 and T6 specimens, respectively.

**Figure 4:**
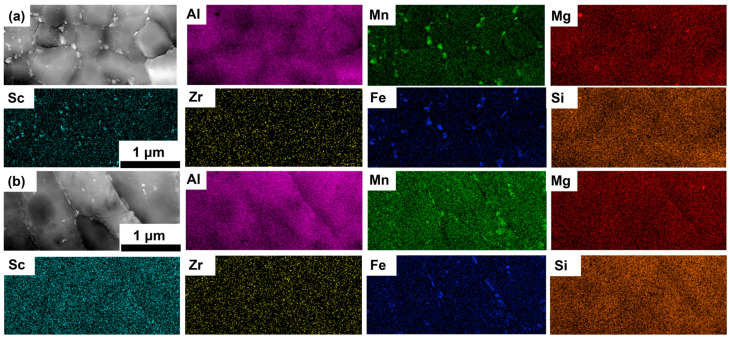
STEM-HAADF images and corresponding EDS maps of the LPBF-fabricated Al–Mn–Mg–Sc–Zr alloy in the as-built condition in the EG (**a**) and CG (**b**) regions.

**Figure 5:**
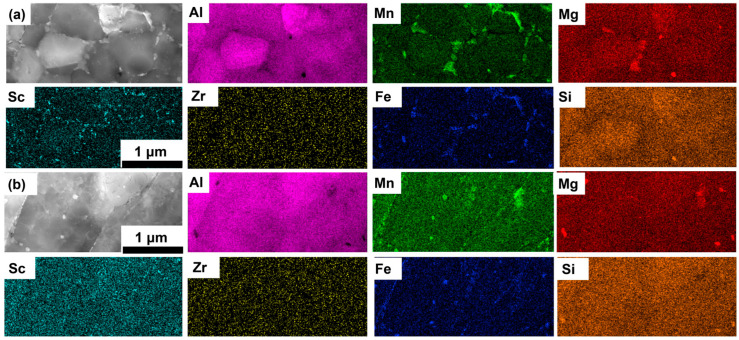
STEM-HAADF images and corresponding EDS maps of the LPBF-fabricated Al–Mn–Mg–Sc–Zr alloy in the T5 heat-treated condition in the EG (**a**) and CG (**b**) regions.

**Figure 6:**
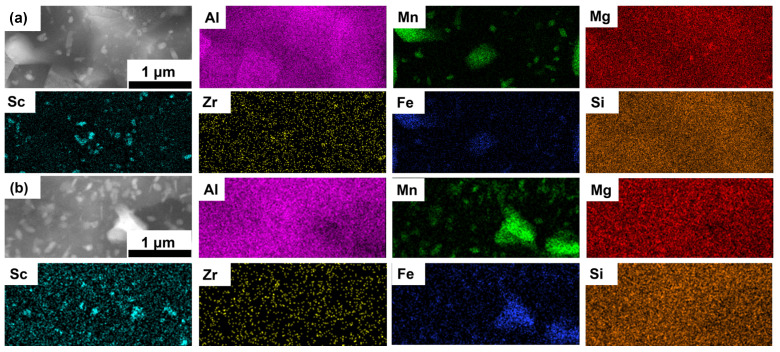
STEM-HAADF images and corresponding EDS maps of the LPBF-fabricated Al–Mn–Mg–Sc–Zr alloy in the T6 heat-treated condition in the smaller EG (**a**) and larger EG (**b**) regions.

Figure 4, Figure 5 and Figure 6 present STEM-HAADF images and the associated EDS elemental maps of the AB, T5, and T6 specimens. In the AB and T5 specimens (Figure 4 and Figure 5), the microstructure revealed three distinct precipitates: (1) many primary Al_3_X (X = Sc/Zr) precipitates (highlighted in cyan) located at the EG region, which were less in CG boundaries; (2) primary Al_6_(Mn, Fe) precipitates (highlighted in green and blue) distributed at the grain boundaries of both EG and CG regions; and (3) Mg_2_Si precipitates (highlighted in red and orange) located at both the EG and CG regions. The morphology and distribution of these precipitates were consistent with previous studies [14,31].

After the T6 heat treatment (Figure 6), significant microstructural changes were observed. The primary Al_3_X precipitates became larger, as shown in the Sc EDS map. There were two types of Al_6_(Mn, Fe): one had a grain size similar to α-Al (primary Al_6_(Mn, Fe)), and the other was finer (secondary Al_6_(Mn, Fe)) and mainly located within α-Al grain interior, while the Mg_2_Si precipitates were partially dissolved.

Table 2 summarizes the sizes of these precipitates after different treatments. The average diameters of the primary Al_3_X precipitates were measured at 51 ± 20 nm, 52 ± 18 nm, and 75 ± 20 nm for the AB, T5, and T6 specimens, respectively. Similarly, the average diameters of the primary Al_6_(Mn, Fe) precipitates were 122 ± 63 nm, 125 ± 57 nm, and 380 ± 257 nm for the AB, T5, and T6 specimens, respectively. For both the AB and T5 specimens, negligible variation was observed in the diameter of the primary Mg_2_Si precipitates (AB: 66 ± 14 nm; T5: 65 ± 12 nm). However, the T6 heat treatment resulted in a reduction in the Mg_2_Si size due to the dissolution of Mg_2_Si precipitates (49 ± 3 nm) in the solution treatment. Furthermore, the average diameter of the secondary Al_6_(Mn, Fe) precipitates in the T6-treated specimens was determined to be 108 ± 48 nm.

**Figure 7:**
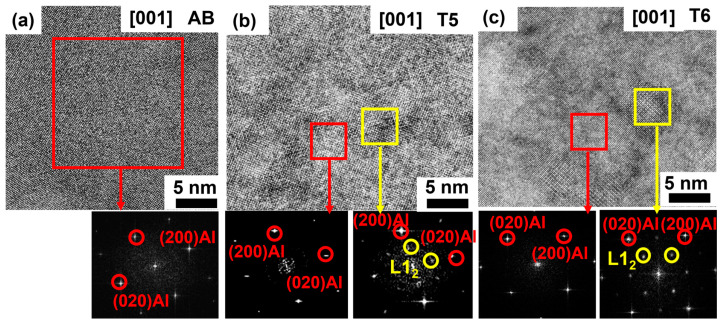
HRTEM images of Al–Mn–Mg–Sc–Zr alloy specimens in the (**a**) AB, (**b**) T5, and (**c**) T6 conditions. The bottom insets show the [001] FFT of the secondary Al_3_Sc nanoparticles and the α-Al matrix corresponding to the red and yellow boxes highlighted regions.

HRTEM was employed to examine the nanoscale microstructures of the AB, T5, and T6 specimens. The associated [001] FFT in Figure 7a indicates that there was only an α-Al matrix in the AB specimen. As shown in Figure 7b, a high density of nano-sized precipitates with an average diameter of 1.9 ± 0.1 nm was observed in the T5 specimen. In contrast, after the T6 heat treatment, the mean size of these nano-precipitates increased to approximately 7.4 ± 0.9 nm, as shown in Figure 7c. The FFT pseudo-diffraction analysis confirmed that these nano-precipitates were secondary Al_3_Sc with an L1₂ crystal structure [18]. The larger particle size observed in the T6 specimen, compared to that in the T5 specimen (Figure 7b), indicates that the secondary Al_3_Sc precipitates coarsened during the T6 heat treatment.

### 3.2. Mechanical Property

**Figure 8:**
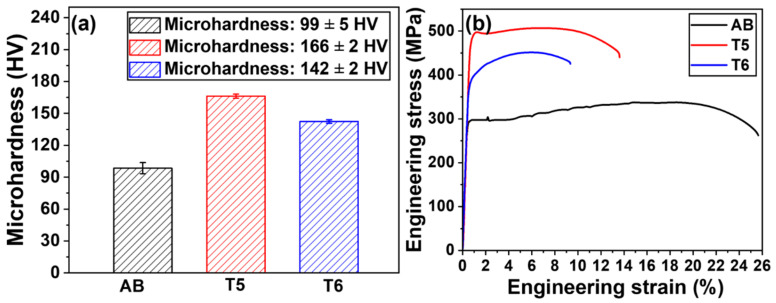
(**a**) Average Vickers hardness of the LPBF-fabricated Al–Mn–Mg–Sc–Zr alloy in different conditions, and (**b**) representative room-temperature engineering tensile stress–strain curves of the LPBF-fabricated Al–Mn–Mg–Sc–Zr specimens in different conditions.

Figure 8a presents the Vickers microhardness test results of the specimens subjected to different heat treatments. According to statistical measurements, the AB specimen had an average microhardness of 99 ± 5 HV. In heat-treated specimens, the T5 heat-treated specimen exhibited an average microhardness of 166 ± 2 HV, which was significantly higher than that of the T6 heat-treated specimen at 142 ± 2 HV.

The representative engineering stress–strain curves of the AB, T5, and T6 specimens are illustrated in Figure 8b. The AB specimen had the lowest strength and the highest elongation at fracture. After heat treatments, the T5 specimen demonstrated higher strength and elongation than those of T6. The trend in tensile strength is the same as that for microhardness. The T5 specimens exhibited a discontinuous yielding behavior with distinct upper and lower yield points: the stress rapidly decreased after reaching the upper yield point, followed by a stable plastic flow stage. This discontinuous yielding phenomenon is closely associated with the insufficient mobile dislocation density within the material [32]. In contrast, the T6 specimen demonstrated a continuous yielding behavior characterized by the absence of a pronounced yield plateau in the stress–strain curve, transitioning directly to the strain-hardening stage. This behavior is attributed to the precipitation-induced homogeneous dislocation multiplication mechanism [33].

In comparison, the LPBF-produced Al–Mn–Mg–Sc–Zr alloys have superior mechanical properties compared to other T5 heat-treated LPBF-produced Al alloys [13]. For T6 heat-treated conditions, the yield strength of the LPBF AlSi10Mg alloy [25] (308 MPa) is 17% lower than that of the LPBF Al–Mn–Mg–Sc–Zr alloy (371 MPa). In addition, the yield strength of the A2024-RAM2 high-strength Al alloy [34] after T6 heat treatment (378 MPa) is comparable to that of the Al–Mn–Mg–Sc–Zr alloy after T6 heat treatment. Therefore, the LPBF-fabricated Al–Mn–Mg–Sc–Zr alloy is known for its high strength.

Table 3 presents the average tensile properties of three replicates for each condition. The AB specimen exhibited the lowest strength but the highest elongation at the fracture, while the T5 condition achieved the best mechanical performance. In contrast, the T6 specimen showed a notable reduction in both strength and ductility compared to T5.

**Figure 9:**
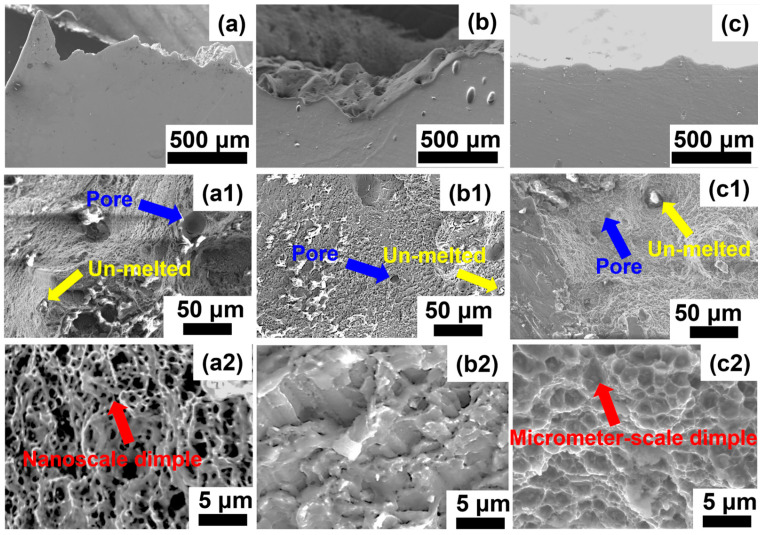
The cross-sectional view and tensile fractured surface of the LPBF-produced Al–Mn–Mg–Sc-Zr alloy in different conditions: (**a**,**a1**,**a2**) AB; (**b**,**b1**,**b2**) T5; (**c**,**c1**,**c2**) T6.

Figure 9 presents the tensile fractured surface of LPBF-fabricated Al–Mn–Mg–Sc–Zr alloy in different conditions. For the AB specimen (Figure 9a), the macroscopic fracture surface exhibited tortuous crack-propagation paths, which indicated superior plastic deformation capability and higher elongation. Microscopic analysis revealed there were a few unmelted particles or pores, and the rest of the region was covered by dimples. The high-magnification image (Figure 9(a2)) demonstrates uniformly distributed nanoscale dimples, which may be responsible for the large elongation at fracture in the AB condition, as shown in Figure 8b.

In contrast to the AB specimen, the T5-treated specimen displayed a less fluctuating macroscopic fracture surface (Figure 9b), which reflected localized non-uniform plastic deformation. Figure 9(b1) shows a few unmelted particles and pores, and the high-resolution image (Figure 9(b2)) reveals typical intergranular brittle fracture characteristics. In a previous study [10], this was attributed to the coplanar slip induced by secondary Al_3_Sc precipitates, which introduced stress concentration at grain boundaries and preferential intergranular embrittlement fracture (Figure 9(b2)). Therefore, the ductility was reduced in the T5 specimen compared to the AB condition.

After the T6 heat treatment, the specimen possessed the lowest ductility, which had a relatively flat macroscopic fracture surface, as shown in Figure 9c. The high-magnification image (Figure 9(c1)) discloses prominent tear ridges and quasi-cleavage fractures, which indicate relatively brittle fractures in T6 compared to the other two conditions. This phenomenon was attributed to the coarsened brittle primary Al_6_(Mn, Fe) and secondary Al_6_(Mn, Fe) precipitates (Figure 6 and Table 2), which induced localized stress concentration at grain boundaries during the tensile process and then triggered microscopic cleavage fractures [35]. Notably, despite the overall brittle fracture characteristics, numerous micrometer-scale dimples are observed in Figure 9(c1,c2), with dimensions significantly larger than the nanoscale dimples in the AB specimen [35]. This might explain why, although the T6 specimen had the lowest ductility elongation in the three conditions, it still had a rational elongation at fracture of 9%.

Compared to the AB specimen, the dispersed secondary Al_3_Sc precipitates in the T5 specimen significantly enhanced the alloy strength, as shown in Figure 8. However, the T6 heat treatment induced a pronounced strength reduction compared to the T5 specimens (Figure 8b and Table 3), which can be attributed to three factors. (1) The microstructural characterization (Figure 3) revealed that during the 500 °C solution treatment in the T6 process, the primary Al_3_X and primary Al_6_(Mn, Fe) precipitates significantly coarsened. The coarsened primary precipitates combined with the partial dissolution of Mg_2_Si precipitates weakened the grain boundary pinning effect, which resulted in significant α-Al grain growth after the T6 treatment, as shown in Figure 3. According to the Hall–Petch relationship [36], an increase in α-Al grain size reduces the contribution of grain boundaries to material strengthening. (2) The coarsening of secondary Al_3_Sc precipitates reduced their coherent strain with the matrix, thereby diminishing their effectiveness in hindering dislocation motion and weakening the precipitation strengthening effect. (3) As reported in a previous study [14], the partial decomposition of the Mn-rich solid solution phase during aging at 450 °C in LPBF Al–Mn–Mg–Sc–Zr alloys led to a reduction in solid solution strengthening. In the present study, the T6 heat treatment included a solution treatment at 500 °C, which would also have contributed to a decrease in the solid solution strengthening. Therefore, the overall reduction in the strength of the T6 specimen was attributed to the reduced grain size strengthening, precipitation hardening, and solid solution strengthening.

## 4. Conclusions

This study investigated the effects of different heat treatments on the microstructure and mechanical properties of an Al–Mn–Mg–Sc–Zr alloy fabricated by LPBF. The key findings and conclusions are summarized as follows:

- After T5 heat treatment, the grains exhibited an alternating distribution of equiaxed and columnar structures, which was similar to the as-built specimen. In contrast, T6 heat treatment induced recrystallization, and the bi-modal structure of columnar and equiaxed grains disappeared. In addition, T6 heat treatment led to significant α-Al grain growth, and the microstructure was mainly an equiaxed grain structure.
- After T5 heat treatment, secondary Al_3_Sc formed, and the yield strength increased as a result of precipitation hardening compared to the as-built condition.
- Compared to the T5 condition, the T6 heat treatment led to significant coarsening of primary Al_3_X and Al_6_(Mn, Fe) precipitates with partial dissolution of Mg_2_Si precipitates. This weakened the grain boundary pinning effect and resulted in larger α-Al grains. Additionally, secondary Al_3_Sc coarsened significantly after T6, which reduced the precipitation hardening effect. As a result, T6 specimens had a lower strength compared to their T5 counterparts.
- The as-built specimens had the highest ductility, and the T5 specimen had a reduced elongation at fracture as a result of precipitation hardening. In addition, the ductility was further decreased in the T6 specimens, which was related to the brittle cleavage caused by the stress concentration effect of coarsened primary Al_3_X, primary Al_6_(Mn, Fe), and secondary Al_6_(Mn, Fe) precipitates.

## Figures and Tables

**Table 1:** Powder properties (wt.%).

Material	Al	Mn	Mg	Sc	Zr	Fe	Si
Al–Mn–Mg–Sc–Zr	Bal.	3.12	1.63	0.69	0.24	0.075	0.054

**Table 2:** The precipitate size (nm) of primary Al_3_X, primary Al_6_(Mn, Fe), secondary Al_6_(Mn, Fe), Mg_2_Si, and secondary Al_3_Sc. The measurements were performed by using an ImageJ 1.8.0 software on STEM-HAADF and HRTEM images.

Specimens	Primary Al_3_X (nm)	Primary Al_6_(Mn, Fe) (nm)	Mg_2_Si (nm)	Secondary Al_6_(Mn, Fe) (nm)	Secondary Al_3_Sc (nm)
AB	51 ± 20	122 ± 63	66 ± 14	–	–
T5	52 ± 18	125 ± 57	65 ± 12	–	1.9 ± 0.1
T6	75 ± 20	380 ± 257	49 ± 3	108 ± 48	7.4 ± 0.9

**Table 3:** Room temperature tensile properties of LPBF produced Al–Mn–Mg–Sc–Zr alloy after T5 and T6 heat treatments.

Specimens	YS (MPa)	UTS (MPa)	*ε_f_* (%)
AB	294 ± 11	337 ± 1	24 ± 2
T5	484 ± 1	507 ± 3	15 ± 1
T6	371 ± 10	442 ± 8	9 ± 1

## Data Availability

The raw data supporting the conclusions of this article will be made available by the authors on request.
